# Somatization, Body Perception, and Pain Processing in Anxiety and Psychotic Disorders

**DOI:** 10.1192/j.eurpsy.2026.10789

**Published:** 2026-07-31

**Authors:** M. Gietka, M. Bibik, J. Czyżewska, A. Kateryna, P. P. Świtaj, J. Dąbrowska

**Affiliations:** 1 Masovian Voivodeship Drewnica SP. Z O.O. HOSPITAL, Ząbki; 2 Psychiatric, Bielanski Hospital, Warszawa, Poland

## Abstract

**Introduction:**

Somatization and altered body perception are emerging as key transdiagnostic dimensions in psychiatry. Somatization reflects the manifestation of psychological distress through physical symptoms that lack a clear organic cause (Xiaoya Fu, 2019). It may be accompanied by disturbed interoceptive awareness—an increased sensitivity to internal bodily signals that promotes maladaptive interpretations of ordinary sensations (Jenkinson, 2024). In anxiety disorders, this manifests as excessive vigilance toward minor bodily cues, amplifying discomfort and maintaining distress. Conversely, psychotic and affective disorders may involve blunted or dysregulated sensory processing, resulting in hypoalgesia and reduced body awareness. However, it remains uncertain whether patients with schizophrenia truly experience less pain, as cognitive deficits and negative symptoms may limit the recognition or expression of pain sensations (Chi, 2022; Urban-Kowalczyk, 2015).

**Objectives:**

This review aimed to synthesize current evidence on somatization, interoception, and pain perception across anxiety and psychotic disorders and to identify shared neurobiological mechanisms underlying altered body representation and sensory processing.

**Methods:**

A literature review was conducted using PubMed, Scopus, Web of Science, and Google Scholar for studies published between 1988 and 2025 in English-language, peer-reviewed journals. Eligible studies included original research, randomized controlled trials, and systematic or narrative reviews focusing on somatization, interoceptive processes, and pain perception in psychiatric populations.

**Results:**

Evidence indicates distinct patterns of bodily processing across psychiatric disorders. Anxiety is consistently linked to somatosensory amplification, first described by Barsky et al. (1988) and later expanded into the concept of “somatic threat amplification” (Köteles & Witthöft, 2017), as well as to an attentional bias toward bodily sensations, resulting in lower pain thresholds and greater symptom severity (Barends, 2020; Scaini, 2025). Moreover, it has been reliably associated with increased negative evaluation and perceived frequency of bodily signals (Clemente, 2024). In contrast, schizophrenia and major depression show reduced pain sensitivity—generalized in schizophrenia and limited to low-intensity stimuli in depression (Kim, 2022). Neuroimaging studies suggest that the insula and somatosensory cortex may play a key role in these differences in interoceptive and nociceptive processing.

**Conclusions:**

Understanding the mechanisms of altered interoception, somatosensory amplification, and dysregulated pain perception is crucial for linking bodily processing with psychopathology. Further interdisciplinary research is needed to elucidate the complex relationships between pain processing and psychiatric symptoms and to develop novel therapeutic approaches.

**Disclosure of Interest:**

None Declared

